# Performance Enhancement of Capacitive-Coupling Dual-gate Ion-Sensitive Field-Effect Transistor in Ultra-Thin-Body

**DOI:** 10.1038/srep05284

**Published:** 2014-06-13

**Authors:** Hyun-June Jang, Won-Ju Cho

**Affiliations:** 1 Department of Electronic Materials Engineering, Kwangwoon University, 447-1, Wolgye-dong, Nowon-gu, Seoul 139-701, Republic of Korea

## Abstract

Recently, thin-film transistor based-ISFETs with the dual-gate (DG) structures have been proposed, in order to beat the Nernst response of the standard ISFET, utilizing diverse organic or inorganic materials. The immutable Nernst response can be dramatically transformed to an ultra-sensing margin, with the capacitive-coupling arisen from the DG structure. In order to advance this platform, we here embedded the ultra-thin body (UTB) into the DG ISFET. The UTB of 4.3 nm serves to not only increase its sensitivity, but also to strongly suppress the leakage components, leading to a better stability of the DG ISFET. In addition, we first provide a comprehensive analysis of the body thickness effects especially how the thick body can render the degradation in the device performance, such as sensitivity and stability. The UTB DG ISFET will allow the ISFET-based biosensor platform to continue enhancement into the next decade.

For more than four decades, the ion-sensitive field-effect transistor (ISFET) sensor has been intensively investigated, as a transducer, because of its advantages of label-free detection, fast response, low cost, easy integration, and compatibility with state-of-the-art CMOS manufacturing technologies^1^,^2^. The ISFET is a potentiometric sensor that was initially designed for the detection of hydrogen ions, and can measure changes in the dielectric-electrolyte interface surface potential (*ψ*_0_). Recently, the use of ISFETs is diverted from the *p*H sensor, to transducers of environmental sensors^3^ and biosensors^4^,^5^,^6^,^7^,^8^, by functionalizing the gate dielectric with a specific membrane or biological elements, like DNA, enzyme, living cell, and antibody-antigen complexes. Among them, the immune-sensor has an inherent impediment in determining bio-molecules that is induced by the Debye screening length^9^,^10^, despite the current need of femtomolar detection for early-stage diagnosis of diseases. At the point, the fundamental sensing margin of standard ISFET confined in Nernst response of 59 mV/*p*H can be one of the causes to decline a signal-to-noise ratio of the transducer, merged with non-ideal effects^11^, such as hysteresis, drift, and temperature stability. Hence, beating the Nernst response and suppression of non-ideal phenomena are strongly required, in light of the fact that the high performance ISFET can be transformed into powerful transducers.

In the meantime, the ISFETs with an additional gate, called as the DG ISFET, have been first proposed, utilizing organic thin-film transistor (TFT)^12^ and silicon nanowire field-effect transistor^13^, in order to beat the Nernst response; the thick bottom and thin front gate in Figure 1 act as the primary and secondary gate of the DG ISFET, respectively. The amplification of the Nernst response is achieved from a capacitive-coupling between the primary and secondary gate. In literature, the platform effectively breaks through the barrier of Nernst response, showing outstanding sensitivity ranging from 220 mV/*p*H to 2.25 V/*p*H^13^,^14^. This amplification could eventually serve to the magnification of small signals from the biological conjugation at the secondary gate, which are often limited by electrolyte Debye screening length.

When it comes to the fact that this platform can be realized with various promising body materials, such as a low-price organic^12^ and metal-oxide semiconductors^14^,^15^, it is obvious that the DG ISFET has a great potential to be transformed as powerful transducers easily compatible with current display industries, as an advanced form of the flexible and transparent biosensor^16^. Simultaneously, in consideration of the silicon-based devices, many challenges remain to be practically useful for precise, selective, and reproducible early-stage diagnosis system, due to their relative unstable channel properties^17^,^18^. In this respect, the DG ISFET platform should be more elaborated and optimized.

In this article, we offer a comprehensive analysis in the body thickness effects that have not yet been identified on the DG ISFET, as one of efforts to optimize the platform. In short, thick body produces non-ideal factors, such as unstable coupling ratio and leakage components, in the capacitive-coupling relationship, rendering serious degradation in the device performance. In order to corroborate the influence, we chose confirmable silicon-on-insulator (SOI) substrate and SiO_2_ membrane, as starting materials to be able to eliminate external factors. Also, we elicited highly guaranteed device stability, as well as a larger amplification from the DG ISFET, by embodying the ultra-thin-body (UTB) into the device. The sensitivity of a 4.3-nm-thick UTB device is increased by more than twice, compared to an 85 nm body device, and greater stability improvements can be made in this geometrical property.

## Results

### Electrical characteristics of the DG MOSFET

**Figure 2:**
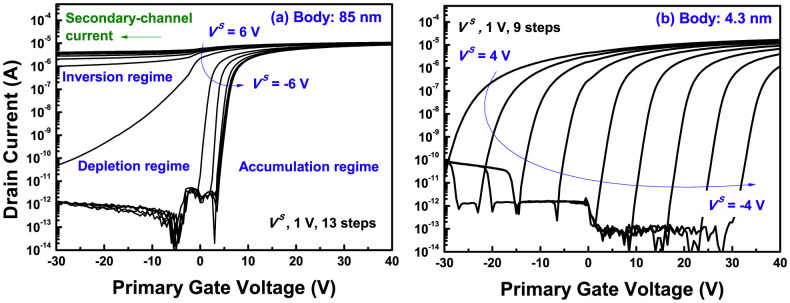
Transfer curves measured by primary gate with the constant secondary gate biases for (a) an 85-nm-thick body, and (b) 4.3-nm-thick body devices. The drain bias was set at 50 mV.

The electric performances of the DG MOSFETs with body thicknesses of 4.3 nm, 30 nm, 61 nm, and 85 nm are evaluated with the secondary gate or primary gate, respectively, at Figure S1. Among them, we chose thicker and thinner body devices, to compare their divergent behaviors in the DG operation. The influence of the secondary gate bias (*V^S^*) on the transfer curves are shown in Figures 2a and 2b, and *vice versa* (Figure S2). According to the classical coupling relation, their systematic interaction between both gates can be described with equations (1) and (2), respectively^19^: 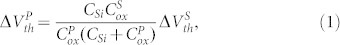


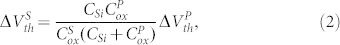
where, 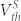 and 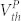 are threshold voltages of a secondary and primary transistor, respectively. 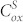, 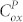, and *C_Si_* are the secondary gate capacitance, the primary gate capacitance, and the depletion capacitance per unit area, respectively. We can express equations (1) and (2) with the physical oxide thickness (POT) term, as following: 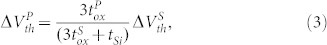


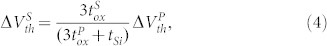
where, 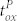, 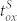 and *t_Si_* are the POT of primary, and secondary gate oxide, and the body, respectively. In principle, this connection is only allowed, where the other channel interface is fully-depleted. Otherwise, the induced inversion or accumulation charges at the other interface screen the transversal electric fields of respective operational gate, and block the systematic interplay among the both transistors. At this point, the body thickness of thin-film transistors becomes a very crucial element to determine the other interface condition, because it directly decides whether the vertical electric fields reach to the other interface or not. In case of the primary gate operation which uses thick bottom gate for a larger 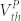 shift, this issue becomes more serious. Hence, in Figure 2a, while the secondary interface becomes either inversion or accumulation by the applied biases, an 85-nm body device reveals saturated or uneven 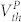 shifts from the induced carriers. The other peculiar point in the plot is the shape of the leakage currents; they are randomly fluctuated with thin secondary inverted carriers^20^; this indicates that parallel shifts do not occur, with the secondary potential changes at regular intervals. In sensor application, they have a negative impact on the linear *p*H response, as discussed below. In contrast, it is obvious that a 4.3-nm body device shows different behaviors: uniform and larger 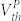 shifts, without leakage currents, all over the operational range. It is attributed to the UTB which imposes a much stronger interface coupling than the normal capacitive-coupling^21^. In the UTB, an entire body is essentially controlled by the primary gate, providing abnormal properties, such as the volume inversion carriers^21^,^22^. Also, it is hard to achieve the secondary accumulation with the negative *V^S^*, due to stronger electric fields arisen from the primary gate^21^. As a result, in the UTB, the device acquires an immunity to the uncontrolled secondary accumulation or inversion carriers, which naturally leads to uniform 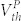 shifts, without leakage components.

**Figure 3:**
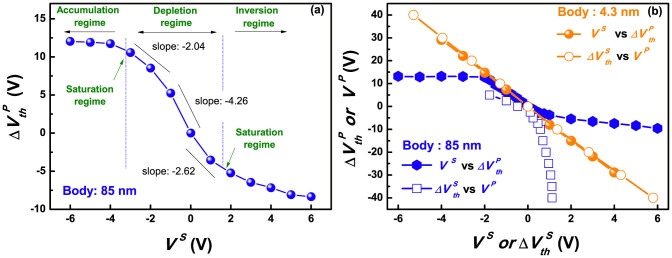
(a) *V^S^* versus 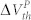 plot of an 85-nm-thick body, extracted from Figure 2a. (b) *V^S^* versus 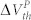 and the reciprocal curve of 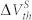 versus *V^P^* of an 85-nm-thick body and a 4.3-nm-thick body devices.

We extracted Figure 3 from the result of Figure 2 to describe the details. The slope at the depletion regime shown in Figure 3a signifies a coupling ratio of an 85-nm thick device, corresponding to 
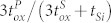
, in equation (3). The value is divergent near the saturation regime from the secondary inverted or accumulated carriers, but allows a solid coupling ratio in the deep depletion region. This means that, for the thick body DG ISFET, the regular amplification cannot be achieved, by the varied coupling ratio, despite even secondary surface potential changes, unless the initial secondary interface state has the deep depletion regime. Unfortunately, the secondary interface is driven closer inversion regime, whiling increasing body, as shown in Figure S1. Meanwhile, the saturated regime disappears in a 4.3-nm-thick UTB, given a constant slope for *V^S^* versus 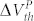 plot, as in Figure 3b. Additionally, the UTB enables the two curves of a superimposed plot of *V^S^* versus 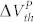 and 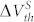 versus the primary gate bias (*V^P^*) to coincide^21^,^22^. In the case of very thin silicon film, the effect of channel capacitance becomes trivial, and each equation (3) and (4) can be reduced with a reciprocal relationship, as follows^23^: 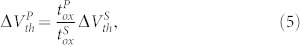


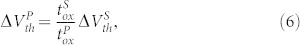
Hence, the reciprocal relation of equation (5) and (6) accounts for the coincidence in the two curves of Figure 3b. Also, it is obvious that the thin body gives a greater coupling ratio than thick body, which leads to a larger shift of 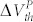 and the amplification in the sensor. The curves of a thick body, however, are incongruent, because they follow the relations of equations (3) and (4), where body thickness influence cannot be ignored.

### Sensing performance of the DG ISFET

A DG ISFET that exceeds the Nernstian response can be realized, grounded in an analogical operation mechanism of the DG MOSFET. The threshold voltage (*V_th_*) of the conventional ISFETs is only adjusted by *ψ*_0_ variation caused by ion interaction, as follows^24^: 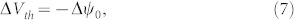
Sequentially, equation (3) can be replaced in the DG ISFET, as: 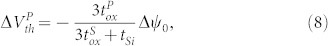
From this relation, the conventional sensitivity is highly amplified by a factor of the capacitive-coupling ratio (
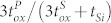
), even though under the DG operation, the Δ*ψ*_0_ is still in the Nernst response. In the paper, we defined the value of 
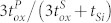
 as the ideal amplification factor (AF) and that of 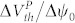 as the experimental AF.

**Figure 4:**
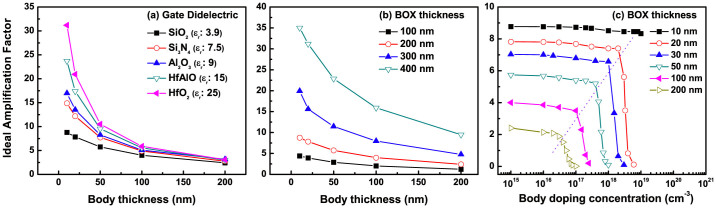
TCAD device (ATLAS, Silvaco™) simulation results of the ideal AF, based on various fabrication elements as a function of body thickness. (a) dielectric constant, (b) BOX, and (c) body doping concentration influence.

We simulated the coupling relationship with various fabrication elements as a function of body thickness, using ATLAS (Silvaco™), and extracted the ideal AF, as shown in Figure 4. The detail simulation conditions are presented in the supporting information. In order to gain a higher coupling ratio, 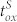 should be thinner, and 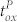 needs to be thicker. Hence, either the use of thin high-k dielectrics at the secondary gate, or the implantation of thicker buried oxide (BOX) is an effective way to gain a greater coupling ratio. Nevertheless, we cannot deny the importance of body influence, because it exponentially enlarges the impacts of the other elements on a coupling ratio, as presented in Figure 4a and 4b. Furthermore, Figure 4c shows that the strong interaction between both gates can be activated, free from the body doping concentrations for the UTB, because of its inherently strong electric fields imposed on the thin channel.

**Figure 5:**
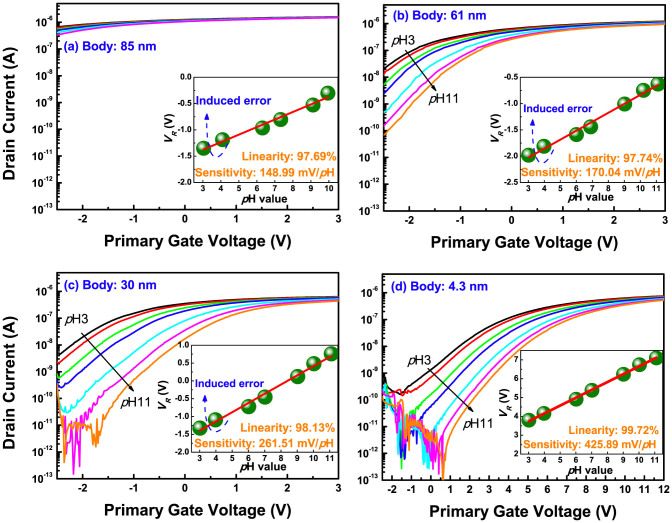
Transfer curves of (a) 85-nm-, (b) 61-nm-, (c) 30-nm-, and (d) 4.3-nm-thick DG ISFETs for the various *p*H buffer solutions. The drain bias is set at 50 mV.

Figure 5 reveals the transfer curves of the DG ISFETs with various body thicknesses, measured in various *p*H buffer solutions. Summaries of the DG and conventional single-gate (SG) sensing performances are shown in Table 1. The responsive voltage (*V_R_*) with *p*H buffer solution is defined as the corresponding the primary gate voltage to the reference currents (*I_R_*). We cannot signify the body effects in the SG operation, because the sensitivity of devices is in a similar distribution from 39.82 mV/*p*H to 47.19 mV/*p*H (Figure S3), within the reported sensitivity of 25 to 48 mV/*p*H^25^. However, for the DG operation, the sensitivity increases, with decreasing body thickness. In particular, a higher sensitivity of 425.89 mV/*p*H is achieved from the UTB device, owing to a larger 
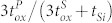
. In aspect of the fact that the sensitivity is increased by more than twice, compared to an 85 nm body device, the body thickness effect cannot be ignored in the DG ISFET platform. Also, it can be noted in Figure 5 that the leakage currents are drastically increased in the thicker body devices, in good agreement with Figure S1 and Figure 2a. This is because the secondary interface gets closer to the inversion regime, with increasing channel thickness; we call this inversion-lopsided coupling.

**Figure 6:**
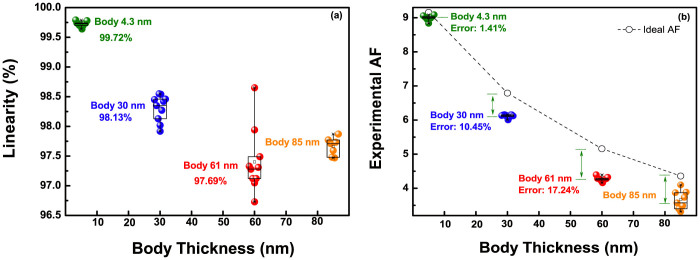
(a) Linearity and (b) experimental AF distribution depending on body thickness, extracted from various *I_R_*.

The inversion-lopsided coupling and leakage fluctuation caused from that give rise to serious errors in the linearity and experimental AF. In order to investigate the impacts on device performance, we extracted the linearity and AFs from various *I_R_*, ranging from 10 nA to 100 nA by 11 steps, as in Figure 6. This analysis gives a clue, whether parallel shift depending on pH occur or not, in light of the fact that the sensing margin is scanned with a wide range of *I_R_* from low to high level. In the case of an 85-nm body device, these parameters were calculated with different *I_R_* range from 900 nA to 1 μA by 11 steps, because of its serious leakage currents. Hence, it is unfair to directly compare the parameters with the other devices. The linearity gradually deteriorates with a wider distribution, on increasing the body thickness: 99.72% (±0.08%) for a 4.3-nm body, 98.24% (±0.32%) for a 30-nm body, and 97.69% (±0.96%) for a 61-nm body. As described in Figure 3a, the thick body gives a varied coupling ratio to the DG ISFET, instead of a solid coupling ratio of the deep depletion regime, especially a smaller coupling ratio near the inversion regime. To be specific, when we measure *p*H solution from base to acid, the coupling ratio in the thick body device, is deceased step-by-step, due to the positive charged ions in the acid solution. In the meantime, the irregular amplification occurs by this varied coupling ratio, which results in the linearity reduction. Thus, in the insets of Figures 5a, 5b, and 5c, the deteriorations in linearity are mainly progressed in measuring the acid solutions.

The influence of leakage fluctuation induced from the inversion-lopsided coupling should be mentioned, in order to account for a wide distribution in the linearity of Figure 6a for the thick body. As shown in Figure 2a, the secondary inverted carriers raise drain currents, randomly, with positive *V^S^*. This behavior spoils a parallel shift of the DG ISFET, and provokes a wide distribution in linearity: a large shift in low *I_R_* level, and a small shift in high *I_R_* level, respectively. In this case, a signal-to-noise ratio can be reduced, for the biosensor application, by triggering a signal fluctuation, especially in real-time measurement: the drain current reading as a function of time under a constant *V^P^*. The UTB that has a strong immunity to leakage components is free from such issues.

Additionally, the thicker body causes larger discrepancy in the values between the experimental AFs (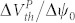) and ideal AFs (
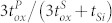
), as shown in Figure 6b. Radically, the ideal AF signifies a solid coupling ratio, at the deep depletion regime. On the other hand, the experimental AF reflects a real coupling ratio; in the process, the thick body devices are given a smaller coupling ratio from the inversion-slanted coupling. Hence, the error between the experimental and the ideal AF gets larger, with increasing body. In contrast, the experimental AF of UTB device is tantamount to the ideal AF, with the lower error value of 1.41%, because the device is inherently given a solid coupling ratio.

**Figure 7:**
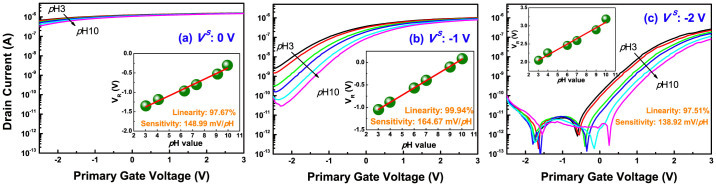
Transfer curves of an 85-nm body device according to applied secondary gate voltage of (a) 0 V, (b) −1 V, and (c) −2 V. The drain bias is set at 50 mV.

**Figure 8:**
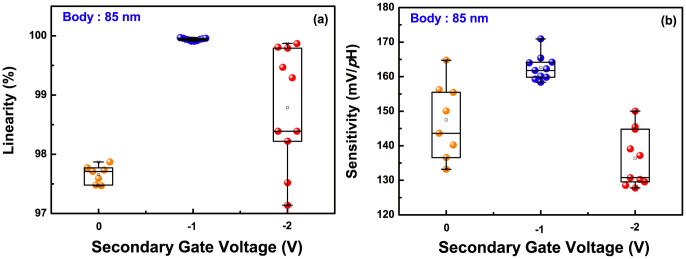
Sensitivity and linearity distribution of an 85-nm body device depending on the negative *V^S^*, extracted from various *I_R_*.

In order to mention the importance of the secondary interface state in the capacitive-coupling, we modified the initial secondary interface of an 85-nm body device near the inversion regime into the deep depletion and accumulation regime, by supplying additional negative *V^S^*, as shown in Figures 7b and 7c. Then, the linearity and sensitivity were extracted with *I_R_*, ranging from 10 nA to 100 nA by 11 steps, and the results are summarized in Table 2 and Figure 8. When applying *V^S^* of −1 V, the leakage currents caused by the secondary inverted carriers are dramatically suppressed, as the initial coupling state is shifted near the deeper depletion regime. Accordingly, the experimental AF (4.1) is contiguous to the ideal AF of 4.36, like as the UTB device of Figure 6b. At the time, the device reveals a better sensitivity, and linearity with a narrow distribution. When we apply more negative *V^S^* of −2 V, the coupling state is switched near the accumulation regime, as in Figure 7c. The parameters are again dispersed, due to the varied coupling ratio. As a result, this shows the inversion- or accumulation-lopsided coupling has a negative on linearity and sensitivity in the DG operation. For better sensing performance, a thin body is highly required in this field.

**Figure 9:**
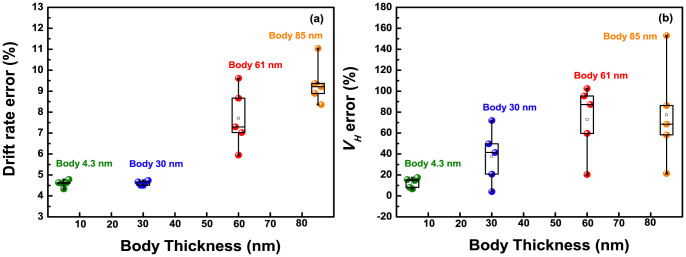
(a) Drift distribution measured by the DG operation in the *p*H 7 buffer solution for 12 hours. (b) Hysteresis distribution for three buffer solutions, measured by the DG operation mode. All evaluations were repeated 5 times.

**Figure 10:**
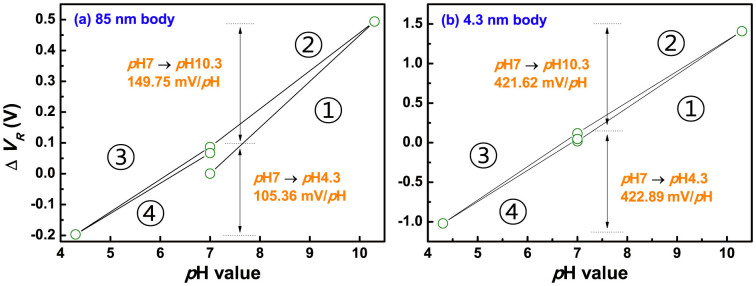
Hysteresis width of (a) an 85-nm body and (b) a 4.3-nm body device, respectively.

General explanations proposed for the non-ideal effects such as hysteresis and drift include several causes: an ion migration enhanced by the electric field within the gate insulator, a penetration of ions from the electrolyte into insulator films, and the slow response surface sites^26^,^27^. These factors bring about a change in the equivalent oxide thickness (EOT) of the sensing membrane or provoke additional *ψ*_0_ variation, and result in the *V_R_* shift of devices^28^. Meanwhile, the capacitive-coupling phenomenon amplifies not only a sensing margin, but also non-ideal effects. In order to compare the degradation degree of devices, exactly, we calculated hysteresis width (*V_H_*) and drift rate errors, from dividing each non-ideal parameter by respective sensitivity in Figure 9. The hysteresis was evaluated, by subjecting the devices to a *p*H loop of 7 → 10 → 7 → 4 → 7, over a period of 60 minutes, and the drift rate was obtained, by long-time monitoring for 12 hours in a *p*H 7. Then, we repeated the tests five times. Likewise with sensitivity and linearity results, the *V_H_* and drift rate errors rise with a wide distribution, on increasing body, as shown in Figure 9. This degradation is also attributed to the inversion-lopsided coupling. In a hysteresis loop of Figure 10a, asymmetric amplified sensing margin is observed from an 85-nm body device, due to a varied coupling ratio, when exchanging a buffer solution from *p*H7 to acid and alkalinity: a larger shift in base and a smaller shift in acid. This negative effect gets serious, combined with further *ψ*_0_ variation caused from the ion damages cause, and the leakage fluctuation that gives random shifts of the drain current. As a result, these comprehensive negative effects provoke additional hysteresis and drift in the thick body. For the UTB device with a solid coupling ratio shown in Figure 10b, such issue can be alleviated, because it is only exposed to ion damages. In conclusion, inversion-slanted coupling state of thick body device has negative impacts on the DG operation, by declining overall device performance at sensitivity, linearity, hysteresis, and drift behavior.

## Conclusion

In this work, the various benefits induced by the UTB are newly recognized in the DG ISFET, by comparing the sensing characteristics of each device with different body thickness. The UTB DG ISFET shows an outstanding sensitivity of 425.89 mV/*p*H, with an enhanced chemical stability. These improvements are attributed to the UTB that gives a stronger coupling and provides various advantages in the DG ISFET platform, such as a larger amplification, a solid coupling ratio, suppression of leakage currents, and elimination of the 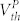 saturation behavior. These effects allow the DG ISFET to have all-round reinforcements in sensitivity, linearity, drift and hysteresis characteristics. This is especially important, given the fact that the development of high performance ISFETs is directly related to the feasibility of a high quality biosensor in future applications. Also, the innate strong electric fields on the UTB can reinforce the unstable channel characteristics of the DG ISFETs, based on the other organic or inorganic channel, by suppressing leakage components, and by increasing their signal-to-noise ratio. Therefore, the UTB DG ISFET platform will serve as one of the key motivators to train next generation ISFET-based biosensors, extending their potentials over manifold fields, such as biomedical diagnostics, drug discovery, environmental, and process monitoring systems.

## Methods

The DG ISFETs devices were fabricated on fully depleted p-type (100) SOI substrates with a doping concentration of 1 × 10^15^ cm^3^, a 107-nm-thick top silicon layer and a 224-nm-thick BOX. In order to produce the UTB, the top silicon layers were etched using a 2.38% tertramethylammonium hydroxide (TMAH) solution (Etching rate = 3.294 nm/min at 25°C), for precise thickness control. The surface roughness of the top silicon layer after TMAH etching, measured by atomic force microscopy (AFM), was less than 0.17 nm. In forming ultra-thin-film, uniform thickness is highly required, because the threshold voltage of the device can be largely dispersed with small roughness difference. The designed active channel length and width of the DG ISFETs are 10 μm and 10 μm, respectively. Phosphorus-doped poly-Si layers with a thickness of 100 nm were subsequently deposited at the source/drain (S/D) region, using low-pressure chemical vapor deposition (LPCVD). A SiO_2_ layer with a thickness of 23 nm was grown by thermal oxidation, for the sensing membrane. The cross-sectional TEM image of a 4.3-nm thick body device is shown in Figure S4. Both the UTB and SiO_2_ layer are uniformly etched and grown, respectively. Then, a rapid thermal annealing was carried out at 850°C for 30 s in N_2_ gas ambient, for activation of dopant at the S/D region. The interface charge densities (*D_it_*) between grown SiO_2_ and silicon interface is 1.46 × 10^10^ extracted from the low- and high-frequency capacitance curves of metal-oxide semiconductor (MOS) capacitors (Figure S5). After deposition of the aluminum layer with a thickness of 150 nm for S/D contacts by an e-beam evaporator system, a forming gas annealing at 450°C for 30 min in a 2% H_2_/N_2_ ambient was carried out, to improve the electrical properties of the ISFETs. The reservoir for injection of *p*H buffer solutions was formed from polydimethylsiloxane (PDMS), and the ISFETs were immersed in reversed osmosis water for 12 hours, to detect steady *p*H responses. The current versus voltage curves for various *p*H buffer solutions were measured, using commercial Ag/AgCl reference electrodes connected to a Hewlett-Packard 4156B high-precision semiconductor parameter analyzer, in a shielded dark box at room temperature, to protect from light and noise interference.

## Author Contributions

H.-J.J. and W.-J.C. wrote the main manuscript and H.-J.J. prepared Figure 1–10 and Table 1–2. All authors reviewed the manuscript.

## Additional information

**Additional Information** Supplementary Information describes transfer curves of MOSFETs measured by the primary and secondary gate, sensitivity of the DG ISFETs measured by the SG mode, TEM image of device, and the interface charge densities.

## Supplementary Material

Supplementary Information Supporting information

## Figures and Tables

**Figure 1:**
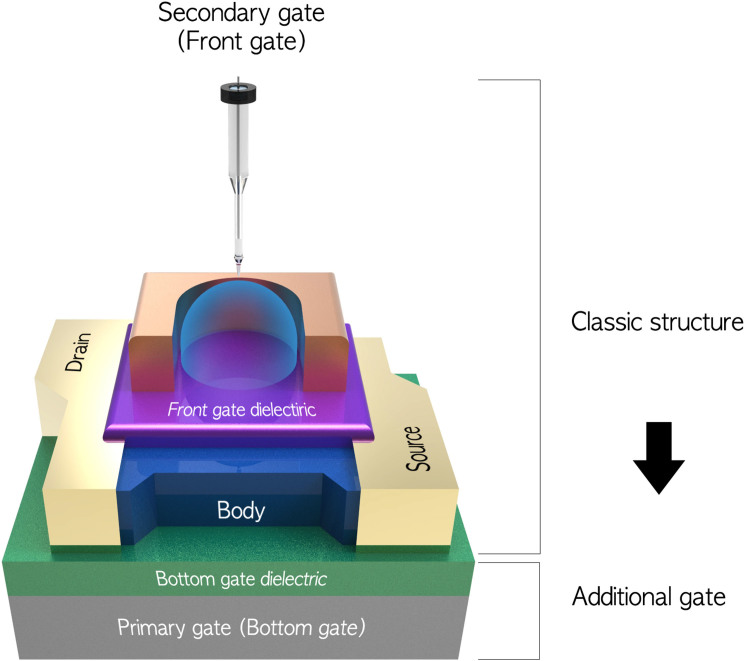
A cross-sectional schematic image of the DG ISFET.

**Table 1:** Sensing properties of the DG ISFETs with various body thicknesses extracted from the DG or SG measurement

	Sensitivity (mV/*p*H)		Linearity (%)		Drift error (%)	V_H_ error (%)	Amplified factor	
Body Thickness (nm)	SG	DG	SG	DG	DG	DG	Expri.	Ideal
85	40.19	148.99(±15.77)	99.4	97.67(±0.2)	9.7(±1.34)	87.13(±65.83)	3.71	4.36
61	39.82	170.04(±4.65)	99.14	97.69(±0.96)	7.78(±1.83)	61.5(±41.12)	4.27	5.16
30	43.1	261.51(±2.72)	99.13	98.24(±0.32)	4.62(±0.12)	38.14(±33.96)	6.08	6.79
4.3	47.19	425.89(±2.88)	99.72	99.72(±0.08)	4.56(±0.23)	12.01(±5.45)	9.03	9.16

**Table 2:** Sensing properties of an 85-nm body DG ISFET according to negative *V^S^*

*V^S^* (V)	Sensitivity (mV/*p*H)	Linearity (%)	Experi. AF
0	148.99(±15.77)	97.67(±0.2)	3.71
−1	164.67(±6.26)	99.94(±0.03)	4.1
−2	138.92(±11.12)	98.51(±1.36)	3.46
