# Supplementary material for: Performance Enhancement of Capacitive-Coupling Dual-gate Ion-Sensitive Field-Effect Transistor in Ultra-Thin-Body
Source: Sci Rep. 2014 Jun 13;4:5284. doi: 10.1038/srep05284 (PMC4055887; doi:10.1038/srep05284)
Supplement: Supplementary Information — Supporting information [file srep05284-s1.doc]

Performance Enhancement of Capacitive-Coupling Dual-gate Ion-Sensitive Field-Effect Transistor in Ultra-Thin-Body

Hyun-June Jang, and Won-Ju Cho*

Department of Electronic Materials Engineering, Kwangwoon University, 447-1, Wolgye-dong, Nowon-gu, Seoul 139-701, Republic of Korea.

**Supplementary Figure S1.** Transfer curves of SOI MOSFETs with different body thicknesses measured (a) by the secondary gate, and (b) by the primary gate, with each opposite gate grounded, respectively. The inset of Figure S1 (b) shows transconductance depending on the secondary gate voltage, for various body thicknesses measured by the primary gate, with a grounded secondary gate. The drain bias is set at 50 mV.

Figure S1 (a) illustrates the transfer curves of SOI DG MOSFETs with body thicknesses of 4.3 nm, 30 nm, 61 nm, and 85 nm, measured by the secondary gate sweep under the grounded primary gate. The devices represent excellent electrical characteristics, with steep subthreshold swing (SS) values less than 78 mV/dec, and high on/off current ratios larger than 10^8^. Their electrical performances are summarized in Table S1. The SS values for each device are gradually improved, while thinning the body thickness, with the exception of the 4.3-nm-thick UTB device. The stronger electric fields confined in thinner semi-infinite silicon enable a steep SS to be exhibited; but when the body thickness becomes a few nanometers, the back interface traps simultaneously can affect the SS. In principle, the SS in the SOI MOSFET is also determined by the capacitive-coupling relationship, especially between the primary gate capacitance, secondary gate capacitance, and silicon capacitance.^1-3^ When the silicon capacitance becomes negligible in the UTB, naturally, the influences of the primary and secondary gate capacitance are enlarged in the SS of the device. This means that the negative effects arisen from numerous traps in each gate dielectric also get serious. In case of the SOI substrates, they should incorporate numerous back interface traps in its production process, which resulted in a slight degradation of the SS of Figure S1 (a) in the UTB. Also, the threshold voltage of a secondary transistor in the UTB is apparently increased by the quantum confinement effects associated with the quantization of energy, and reduction of the electron density of state at the top silicon layer.^4,5^ Figure S2 (b) shows the transfer curves of the DG MOSFETs measured by the primary gate sweep with grounded the secondary gate. It is hard for the primary gate bias to control the entire channel for a thick body. Hence, the leakage current increases, especially in an 85-nm-thick body. In this case, a very thin front inverted charge or a weak inversion is built up, and leads to that leakage current.^6^ Also, the front inverted charges provoke a secondary peak of transconductance curve,^7^ as shown in the inset of Figure 1S (b). However, when reducing the body thickness, these peaks are steadily suppressed, as the front interface is gradually driven into a deep depletion regime.

Table S1. Electrical characteristics of the SOI MOSFETs measured by the FG operations with a grounded BG.

| Body  thickness (nm) | $V_{th}$ (V) | SS  (mV/dec) | Maximum  on/off current ratio |
| --- | --- | --- | --- |
| 85 | -0.545 | 78 | 2.59 x 10^9^ |
| 61 | -0.426 | 71 | 7.7 x 10^8^ |
| 30 | -0.233 | 63 | 5.4 x 10^8^ |
| 4.3 | 0.674 | 73 | 1.71 x 10^8^ |

**Supplementary Figure S2.** The influence of the primary gate bias on the transfer curves measured by the secondary gate with a 4.3-nm-thick (a) and an 85-nm-thick body (b).

**Supplementary Figure S3.** Transfer curves of the DG ISFETs with different body thicknesses measured by the secondary gate mode.

**Supplementary Figure S4.** (a) Capacitance versus voltage curve of 23-nm-thick SiO_2_ dielectric measured by the low- and high-frequency. (b) The interface charge densities (D_it_) between grown SiO_2_ and silicon extracted from capacitance curves of MOS capacitors.


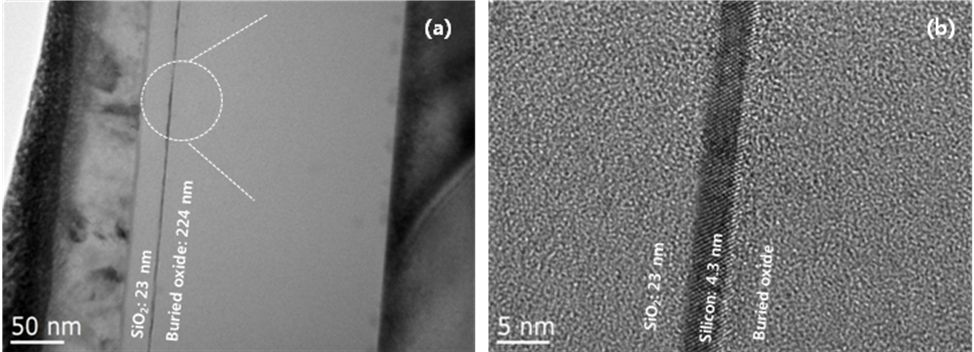


Supplementary Figure S5. A cross-sectional TEM image of the SiO_2_ sensing membrane of 23 nm and a 4.3-nm-thick UTB. TEM analysis was conducted on a JEOL JEM-3010 TEM operating at 300 kV.

**Simulation condition**

Figure 4a shows the body thickness effects on the amplification factor, as a function of the high-k dielectric constant. Their physical oxide thickness (POT) of sensing membrane and buried oxide layer (BOX) is fixed to 20 nm and 200 nm, respectively. Herein, the variables are the dielectric constant of sensing membrane and body thickness in the simulated device. Also, Figure 4b is simulated, to describe BOX effects on the amplification factor. Here, SiO_2_ layer is used as a model membrane, and its POT is fixed to 20 nm. Final simulation of Figure 4c is performed, to determine doping concentration effects on the amplification factor. They modeled device has a 20-nm-thick SiO_2_ sensing membrane, and a 200-nm-thick BOX.

**Supporting Information Reference**

1. Wouters, D. J., Colinge, J.-P. & Maes, H. E., Subthreshold slope in thin-film SOI MOSFET’s IEEE Trans. Electron Devices **37**, 2022-2033 (1990).
2. Mazhari, B., Cristoloveanu, S., Ioannou, D. & Caviglia, A. L., Properties of ultra-thin wafer-bonded silicon-on-insulator MOSFET’s. Trans. Electron Devices **38**, 1289-1295 (1991).
3. Balestra, F., Benachir, M., Brini, J. & Ghibaudo, G., Analytical model of subthreshold swing and threshold voltage for thin- and ultra-thin-film SOI MOSFET’s. IEEE Trans. Electron Devices **37**, 2303-2310 (1990).
4. Majima, H., Saito, Y. & Hiramoto, T., Impact of quantum mechanical effects of design of nano-scale narrow channel n- and p-type MOSFETs. in Int. Electron Devices Meeting Tech. Dig. **01**, 733-736 (2001).
5. Omura, Y., Horiguchi, S., Tabe, M. & Kishi, K., Quantum-mechanical effect on the threshold voltage of ultra thin-SOI nMOSFET’s. IEEE Electron Device Lett. **14**, 569-571 (1993).
6. Colinge, J.-P. Transcoductance of silicon-on-insulator (SOI) MOSFET’s. IEEE Electron Device Lett. **EDL-6**, 573-574 (1985).
7. Burignat, S. *et al*. Substrate impact on threshold voltage and subthreshold slope of sub-32 nm ultra thin SOI MOSFETs with thin buried oxide and undoped channel. Solid State Elecronics **54**, 213-219 (2010).
